# Editorial: Impact of microbiome on gut mucosal immunity in health and disease

**DOI:** 10.3389/fimmu.2022.1005156

**Published:** 2022-09-20

**Authors:** Oscar G. Gómez-Duarte, Pearay L. Ogra

**Affiliations:** Division of Pediatric Infectious Diseases, Department of Pediatrics, Jacobs School of Medicine and Biomedical Sciences, University at Buffalo, State University of New York, Buffalo, NY, United States

**Keywords:** gut, immune response, microbiome, bacteria, inflammation

Early studies on immunoglobulin A, the very first identified marker of adaptive immunity in the gut mucosa, quickly led the way to the constantly expanding research field of mucosal immunology. In parallel, gut microbiome, initially studied using conventional microbiology techniques, has expanded with metagenomics technology and metabolomics to uncover an unprecedented amount of information on multiple factors and regulatory pathways linking and modifying the gut immune system as well as distant organ-systems including the central nervous system. The human gut has an abundant microbial load of bacterial genus, representing >2,000 microbial species (>160 species/individual). It is estimated that for every human cell in the body, there may be as many as 1-10 bacterial cells resident in mucosal surfaces (1). These interactions between the gut’s immune system and microbiome are established from early postnatal development to later stages in life to maintain a homeostatic status but also to allow the host confront the challenges imposed by infectious and non-infectious diseases. Bacteria, fungi, and viruses, major components of the gut microbiome heavily influence mucosal immunity in the mammalian host. Gut and respiratory pathogens continue to challenge the human host with mild to lethal infections. The effect of microbiome on the development of a healthy and robust gut immune response is critical for survival as demonstrated by immune response studies conducted on germ-free animal models and other systems (2). Similarly, microbiome’s influence on gut immune response contributes directly or indirectly to inflammatory disorders and/or autoimmune conditions in the susceptible host.

In this Research Topic, a collection of studies using cutting-edge technology provides information on how microbiome influences gut mucosal immune responses in the healthy host and in hosts affected by inflammatory conditions. Other studies explore how modifications to the microbiome or its metabolic products have a potential as therapeutic agents in the control of intestinal or even extra-intestinal conditions.

Development of a healthy gut immune response at early age is affected by multiple factors including prematurity. Lemme-Dumit et al. provided evidence of three distinct microbiota types differentially associated with intestinal permeability, maternal breastfeeding, and immunological profiles among less than 33 week gestation early preterm infants. The *Staphylococcus epidermidis*, and *Enterobacteriaceae*-predominant microbiota types were associated with increased intestinal permeability, reduced breastmilk feeding, and less defined fecal cytokine profile. On the other hand, a lower intestinal permeability was associated with increased levels of fecal IL-1alpha/beta and a microbiota type that included a wide array of anaerobes with expanded fermentative capacity. These findings may explain, at least in part, the role of intestinal permeability in the pathogenesis of necrotizing enterocolitis in premature infants. Amenyogbe et al. evaluated bacterial and fungal fecal microbiota in rural Ghanaian children and found that bacterial communities differed systematically across the age spectrum in composition and diversity. Corresponding maternal fecal and breastmilk microbiotas had a dramatic change in the maternal postpartum microbiota, including abundance of *E. coli* and lower proportion of *Prevotella* in the first compared to the fourth week postpartum. The dynamics of mother’s bacterial microbiota at the time of birth may have important consequences for children’s health. Sun et al. described the possible negative effects of prenatal maternal stress on intestinal development, impaired barrier function, and gut dysbiosis in a murine model. They also considered the possible role of persistent overgrowth of *Desulfovibrio* in the offspring and its associated colitis in adulthood.

Microbial metabolites generated by individual bacterial species, a collection of different species, or as a result of the host-microbiome virtually countless metabolite combinations, have the ability to influence gut structure and function in many different ways. In this Research Topic, Kaya et al., reported on the metabolite-sensing G-protein coupled receptors (GPCRs) ability to bind to metabolites and trigger signals important for cell function. These receptors provide a link between immune system, gut microbiota, and metabolic system and some GPR35 gene variants are even implicated in gut-related diseases such as inflammatory bowel disease. Different effect of metabolites in the intestine was reported by Deng et al., the gut metabolite pravastatin attenuated intestinal ischemia/reperfusion injury by stimulating IL-13 secretion from type II innate lymphoid cells *via* IL-33/ST2 signaling, a novel mechanism that may have a potential as therapeutic use in cases of intestinal ischemia/reperfusion injury.

In addition to the effect of gut microbiome on the gut immune system, it is fascinating the effect it has in homeostasis of distant organs systems. One review by Bhardwaj et al, described how gut microbiome, prebiotics, and probiotics participate in the regulation of host bone homeostasis, and on the contrary, how dysbiosis of the gut microbiota may lead to bone disease, including osteoporosis. Yu et al., reported on how intestinal microbiome among hospitalized patients in vegetative state versus minimally conscious state significantly differs and how small-chain fatty acid’s production due to this differential bacterial presence were reduced in vegetative state patients compared to other groups.

Authors also explored the potential therapeutic use of probiotics and other bacterial organisms on gut disease conditions. Fidanza et al. reviewed *Lactoplantibacillus platanarum* and its ecological and metabolic flexibility allowing it to thrive in multiple environments and making it an probiotic with potential applications for treatment or prophylaxis of a variety of disorders. Gong et al. found that *L. plantarum* and *Paenibacillus polymyxa* given to one-day chicks protected them against *Clostridium perfringens* infection through improved composition and metabolic pathways of the intestinal microbiota, intestinal structure, inflammation, and anti-apoptosis. Furthermore, Xu et al. reported on how probiotic mixtures significantly alleviated skin inflammation of chemically-induced atopic dermatitis in mice by increased production of regulatory T cells and regulatory dendritic cells in mesenteric lymph nodes. Finally, Cait et al., provided evidence on how a high fiber diet and the resulting short-chain fatty acid metabolites are associated with enhanced antibody response to seasonal influenza vaccination.

The elegant studies presented in this Research Topic using cutting-edge technology allow us to better understand the mechanisms by which gut microbiota and their metabolites affect gut immune responses as well as systemic immune responses to intrinsic and extrinsic antigenic stimuli during early or late development. It also provides evidence of microbial and metabolic signatures associated with a variety of host states of health or disease conditions. Furthermore, it provided evidence on how gut immune system modifiers, such as microbial organisms, food products and metabolites may have therapeutic potential to a variety of conditions. Regulating gut microbiota and the intrinsic and extrinsic metabolomics it may be possible to control gut immune response locally or distally. Finally, each area of research exploring the relationship between gut microbiome and gut immune system in health and disease keep uncovering more questions than answers that should be the basis for novel and exciting research in the near future.

## Author contributions

Both authors made substantial contributions to the conception and design of the editorial. OG-D wrote the first draft of the manuscript and both, OG-D and PO, extensively and critically revised the draft and added important intellectual content. Once both authors were satisfied with the final draft, they provided approval for publication.

## Conflict of interest

The authors declare that the research was conducted in the absence of any commercial or financial relationships that could be construed as a potential conflict of interest.

## Publisher’s note

All claims expressed in this article are solely those of the authors and do not necessarily represent those of their affiliated organizations, or those of the publisher, the editors and the reviewers. Any product that may be evaluated in this article, or claim that may be made by its manufacturer, is not guaranteed or endorsed by the publisher.

## References

[1] Gómez-Duarte OG Ogra PL. Development of mucosal immunity: Functional interactions with mucosal microbiome in health and disease. CIR (2019) 15:154–65. doi: 10.2174/1573395515666190225153529

[2] Hooper LV Wong MH Thelin A Hansson L Falk PG Gordon JI. Molecular analysis of commensal host-microbial relationships in the intestine. Science (2001) 291:881–4. doi: 10.1126/science.291.5505.881 11157169

